# Sleep Deprivation Therapy Enhanced Via Repetitive Transcranial Magnetic Stimulation in Major Depression

**DOI:** 10.7759/cureus.2174

**Published:** 2018-02-08

**Authors:** Abhishek Gupta

**Affiliations:** 1 Geriatrics, Center for Addiction and Mental Health

**Keywords:** rtms, transcranial magnetic stimulation, sleep deprivation, depression

## Abstract

Transcranial magnetic stimulation (rTMS) and sleep deprivation (SD) are two of the latest advances made in the field of psychiatric research. Though yet in development, they present unique opportunities to achieve significant clinical outcomes particularly in major depression disorder (MDD). A limited set of studies have been done in the combined use of rTMS-SD in treating MDD. While promising, these studies have been hampered by the limited knowledge of rTMS and SD themselves due to their relatively recent use as viable therapeutic options. This review is aimed at an analysis of the limitations observed in the studies conducted to date involving rTMS and SD. In addition, it explores the potential new avenues for future research in the deployment of rTMS-SD as a viable treatment option.

## Introduction and background

Depression is a major psychiatric epidemic affecting more than 350 million individuals worldwide [1]. As such, the treatment of major depression has been a major frontier in psychiatric research, traditionally involving both pharmacological and non-pharmacological options. Non-pharmaceutical options have usually included psychotherapy and electroconvulsive therapy [1]. However, recent advancements at the nonpharmacological frontier have given rise to new options for clinicians in order to achieve better clinical outcomes. Sleep deprivation therapy and transcranial magnetic stimulation (rTMS) are some of these new options.

Sleep deprivation (SD) is a methodology developed in the 1970s when antidepressant effects were observed in controlled sleep deprivation in individuals. Two major forms of SD are total and partial sleep deprivation. In total SD therapy, a patient is prevented from sleeping for a complete 24-hour cycle whereas partial SD therapy allows for only first half of a sleep cycle to be completed [1]. SD alters neurological networks associated with depressed individuals in three areas. The first, the default mode network (DMN), comprises of midline frontal cortex, inferior parietal lobule, and the precuneus areas. The second, cognitive control network (CCN), includes bilateral fronto-cinguloparietal areas, especially the lateral prefrontal and superior parietal areas. The third, perhaps the most implicated network in depressed individuals, is the affective network (AN). AN includes subgenual and pregenual areas, including Brodman area 32. Abnormal neurological activity between these three brain networks and the dorsomedial prefrontal cortex (DMPFC) has been found in depressed patients compared to healthy individuals. These areas with abnormal activity are collectively called the dorsal nexus (DN) and are suspected to be the neurological focus of mood dysregulation [2]. As such, DN has become a new focus for psychiatric treatment in order to resolve clinical depression [1].

Transcranial magnetic stimulation uses focal magnetic waves to stimulate specific brain areas whose under-activity has been associated with neural dysregulation. Unlike traditional electroconvulsive therapy (ECT), rTMS does not require general anesthesia or seizure prophylaxis [3]. Secondly, ECT is known for causing prolactin release while rTMS does not. Lastly, ECT causes widespread hypothalamic pituitary adrenal (HPA) axis stimulation instead of the more specific increased cortisol secretion observed in depressed patients receiving rTMS [4]. As such, rTMS has significant advantages in treating depression and other psychiatric disorders compared to traditional technologies such as ECT.

## Review

Combined intervention

rTMS has been theorized as a viable way to prolong the antidepressant effects of SD [5]. Eichhammer et al. [6] showed that the antidepressant effect of partial SD can be prolonged up to four days, effectively preventing relapses in post-SD therapy recipients. Krstić et al. [7] observed the same prolonged antidepressant effect in partial SD recipients for up to six months. Additionally, Luber et al. [8] showed that rTMS could prevent cognitive deficits associated with sleep deprivation. This opens a new frontier in SD co-treatment as not just a way to prolong the antidepressant effect of SD, but also to prevent any impairment in working memory tasks caused by SD [8]. This combined approach is hypothesized to synergistically modulate the neuron networks in the anterior cingulate cortex, also considered to be involved in depression pathophysiology. To date, three studies have been performed on this combined intervention approach [1] (Table 1). Of these three, two have shown an augmented antidepressant effect from SD via rTMS while the remaining study showed no significant improvement in clinical outcomes [5]. Therefore, further research is warranted in the potential use of rTMS-SD.

**Table 1:** Comparison of studies performed on rTMS-SD combined treatment *DSM-IV*: Diagnostic and Statistical Manual of Mental Disorders, 4th Edition; *MINI*: Mini-International Neuropsychiatric Interview Criteria; *Exp*: Experimental group; *Ctr*: Control group; *TSD*: Total sleep deprivation; *PSD*: Partial sleep deprivation; *rTMS*: Repetitive transcranial magnetic stimulation; (*-*): No report in article; *DLPFC*: Dorso-lateral pre-frontal cortex; *Hz*: Frequency; *MT*: Motor threshold table Adapted from Tang et al. [1]

Study	Number of Subjects (Exp/Ctr)	Mean Age, Y (Exp/Ctr)	Treatment Regimen	Primary Diagnosis	Diagnostic Criteria	Coil/Position	Frequency (Hz)	Intensity (% of MT)	Sessions
Eichhammer et al. (2002) [6]	20 (10/10)	46.7 (44.9/48.5)	PSD + rTMS	15 Major Depression; 5 Bipolar-1	DSM-IV	Figure-eight shaped/ Left DLPFC	10.0	80	4
Kreuzer et al. (2012) [5]	37 (21/16)	43.0 (45.3/39.9)	TSD + rTMS	Acute Depression	DSM-IV	Figure-eight shaped/ Left DLPFC	10.0	110	4
Krstić et al. (2014) [7]	19 (11/8)	48.3 (-/-)	PSD + rTMS	Unipolar Major Depression	DSM-IV and MINI	Figure-eight shaped/ Right DLPFC	1.0	110	10

Protocol review

To date, studies conducted on rTMS have focused on acute unipolar or bipolar depression [9]. Enrollment protocols primarily disallow any use of tricyclic antidepressants or neuroleptics due to potential seizure risk [3]. In addition, studies in SD have screened participants based on a history of substance abuse, epilepsy, stroke, and fluctuations in antidepressant medication levels [1, 3]. Further, the same three trials mentioned above only focus on the dorsolateral prefrontal cortex (DLPFC). As such, many of the neural foci suspected to be culprits in depression neuro-pathophysiology discussed above (DMN, AN, CCN) have not been fully investigated. Lastly, the mean age of participants in all studies conducted to date was 40 years [1].

Limitations

Both SD therapy and rTMS carry risks in their application. At first, both can be used currently as adjunctive treatments only, as part of a greater treatment regimen.

SD, especially the classic variant of total deprivation, has a rapid but limited duration of effect [1, 10]. Partial SD, especially, often shows quick relapses into depression after a single night of recovery sleep [6]. Attempts to prolong this antidepressant effect in bipolar patients by combining sleep deprivation with lithium carbonate, advanced sleep phase or bright-light therapy have shown some promise [11]. However, such combination therapy based on multiple circadian interventions remains at the experimental stage. Also, to date, SD trials have been conducted in an inpatient setting without a double-blind format given that all SD recipients inherently know of being deprived of sleep [11]. This is a significant drawback in any scientific experiment. Logistically, it also represents a significant challenge for any application of SD therapy in an outpatient setting.

rTMS, on the other hand, has had numerously reported side effects in trials. Primarily, seizures are a major concern based on reported cases in trials. It is hypothesized that rTMS may induce the 'kindling' phenomenon, thereby reducing the seizure threshold. However, rTMS trials at lower frequencies have significantly avoided the same seizure episodes. As such, using a low-frequency rTMS as well as a thorough screening for prior neurological disorders, predisposing medications, and other seizure risks may considerably limit the risk of seizures in a rTMS recipient. Other potential risks of rTMS include headache, scalp burns, heating of implanted medical devices, and mania [3].

Future investigations

Combined rTMS/SD therapy research can be expanded in multiple directions, including addressing the limitations discussed above. At first, future trials may consider studying neural networks other than just DLPFC. Secondly, comparing the efficacy of total SD and partial SD may yield the ideal combination of two interventions. Additionally, the requirement for a pharmaceutical regimen that does not increase the risk for seizures presents a significant limitation in rTMS/SD application. Therefore, rTMS trials should establish safe medication regimens that can be adopted by rTMS-SD as well without risk of seizures. rTMS/SD therapy trials in younger age groups would also be beneficial since, in such age groups, stroke and other medical comorbidities are rarer. Thereby, this combined therapy may have reduced risks in younger age groups.

rtMS/SD must also investigate the best way to achieve the optimum synergistic effect on neural networks. SD therapy has been known to improve connectivity between the DN and the right DLPFC, but also reduces the connectivity between the anterior cingulate cortex and the bilateral anterior cingulate cortices [1]. Its basis lies in correcting neural dysregulation whereas rTMS functions via a primarily stimulatory effect. As such, future trials must involve fMRI (functional magnetic resonance imaging) use to analyze how individual therapies are affecting the neural connectivity related to the DN and the anterior cingulate cortex. rtMS/SD may achieve greater synergy if their combined effect is limited to improving the connectivity of the anterior cingulate cortex to the other areas within DN.

## Conclusions

To conclude, rTMS/SD therapy presents a new avenue for treating unipolar depression. Current investigations have been limited due to the relative infancy of rTMS and SD therapies themselves. Multiple unknown factors in terms of neurophysiology, efficacy, and risks from comorbidities have a significant limiting effect on the potential of rTMS/SD. As such, current psychiatric research must establish safe and effective regimens for rTMS and SD prior to any further investigations into their joint use. Concrete evidence pertaining to the safety of both rtMS/SD can be a definitive step towards developing large-scale trials for their combined use in treating major depression disorder.

## References

[1] Tang Q, Li G, Wang A (2018). A systematic review for the antidepressant effects of sleep deprivation with repetitive transcranial magnetic stimulation. BMC Psychiatry.

[2] Bosch OG, Rihm JS, Scheidegger M (2013). Sleep deprivation increases dorsal nexus connectivity to the dorsolateral prefrontal cortex in humans. Proc Natl Acad Sci USA.

[3] Loo CK, McFarquhar TF, Mitchell PB (2008). A review of the safety of repetitive transcranial magnetic stimulation as a clinical treatment for depression. Int J Neuropsychopharmacol.

[4] Szuba MP, O'Reardon JP, Evans DL (2000). Physiological effects of electroconvulsive therapy and transcranial magnetic stimulation in major depression. Depress Anxiety.

[5] Kreuzer PM, Langguth B, Schecklmann M (2012). Can repetitive transcranial magnetic stimulation prolong the antidepressant effects of sleep deprivation?. Brain Stimul.

[6] Eichhammer P, Kharraz A, Wiegand R. (2002). Sleep deprivation in depression stabilizing antidepressant effects by repetitive transcranial magnetic stimulation. Life Sc.

[7] Krstić J, Buzadžić I, Milanović SD (2014). Low-frequency repetitive transcranial magnetic stimulation in the right prefrontal cortex combined with partial sleep deprivation in treatment-resistant depression: a randomized sham-controlled trial. J ECT.

[8] Luber B, Steffener J, Tucker A (2013). Extended remediation of sleep deprived-induced working memory deficits using fMRI-guided transcranial magnetic stimulation. Sleep.

[9] Brunoni AR, Chaimani A, Moffa AH (2017). Repetitive transcranial magnetic stimulation for the acute treatment of major depressive episodes: a systematic review with network meta-analysis. JAMA Psychiatry.

[10] Svestka J (2008). Sleep deprivation therapy. Neuro Endocrinol Lett.

[11] Wu JC, Kelsoe JR, Schachat C (2009). Rapid and sustained antidepressant response with sleep deprivation and chronotherapy in bipolar disorder. Biol Psychiatry.

